# The usefulness of a three-protein signature blood assay (Mastocheck®) for follow-up after breast cancer surgery

**DOI:** 10.1007/s00432-022-04550-9

**Published:** 2022-12-24

**Authors:** Yumi Kim, Hong-Kyu Kim, Changjin Lim, Sungsoo Kim, Kyung-Guen Ahn, Dong-Young Noh

**Affiliations:** 1 Department of Surgery, CHA Gangnam Medical Center, CHA University School of Medicine, 566, Gangnam-gu, Seoul, Republic of Korea; 2 Department of Surgery, Seoul National University College of Medicine and Seoul National University Hospital, Seoul, Republic of Korea; 3 Bertis Inc., Seoul, Republic of Korea

**Keywords:** Breast neoplasms, Blood proteins, Proteomics

## Abstract

**Purpose:**

Mastocheck®, a proteomic-based blood assay, has been developed for early diagnosis of breast cancer. The purpose of this study is whether Mastocheck® is useful as a postoperative follow-up.

**Methods:**

A total of 255 patients were analyzed. The patients were classified into longitudinal monitoring and recurrence/nonrecurrence cohorts. The longitudinal monitoring cohort consisted of 111 patients. In this cohort, blood analyses were performed three times (before surgery, 8 weeks after surgery, and between 6 months and one year after surgery), and a comparative analysis of the values of Mastocheck® and individual proteins at each time point was performed. The recurrence/nonrecurrence cohort consisted of 144 patients who had been followed up for more than 1 year, and the blood marker values at the time of local recurrence were compared to those of nonrecurrence patients.

**Results:**

In the longitudinal monitoring cohort analysis, in 81 of 111 patients were diagnosed with breast cancer with Mastocheck® and the sensitivity was 73.0%. Of 111 patients in the longitudinal monitoring cohort, 108 had two blood analyses (before and 8 weeks after surgery), and three serial blood analyses were performed on 53 patients. The Mastocheck® value that were in the cancer range of 73.0% (in 81 of 111 patients) of patients before surgery, was within the normal range of 68.5% (in 74 of 108 patients) at 8 weeks after surgery and 88.7% (in 47 of 53 patients) from 6 months to 1 year after surgery. The value of Mastocheck® was significantly decreased after surgery compared to before surgery (*p* < 0.001). In the recurrence/nonrecurrence cohort analysis, the Mastocheck® values were in the cancer range in 38 out of 63 recurrence patients and within the normal range in 66 of 81 nonrecurrence patients (sensitivity of 60.3% and specificity of 80.2%).

**Conclusions:**

Mastocheck® is expected to be used as a blood marker tool to aid in the early detection of recurrence during follow-up after breast cancer surgery.

## Introduction

Breast cancer is the most commonly diagnosed cancer in women (24.2%, i.e., approximately one in four new cancer cases worldwide), and among 185 countries reported in GLOBOCAN 2018, breast cancer was the most common in 154 countries (Bray et al. 2018). Breast cancer is also the second leading cause of cancer deaths in women after lung cancer (Azamjah et al. 2019). Early detection and treatment are of paramount importance in curing breast cancer. With the development of treatment modalities, long-term survival is expected for breast cancer, with a 5-year survival rate of almost 90% (American Cancer Society 2022), so follow-up after treatment is also crucial. The primary purpose of posttreatment follow-up or surveillance is the early detection of disease recurrence, with the presumption that early detection followed by the early initiation of treatment improves patient outcomes (Chopra and Chopra 2014). Guidelines recommend regular follow-up with history, physical examination, and mammography alone, without other routine laboratory or imaging studies (NCCN 2020). In actual clinical practice, however, many clinicians feel that only history, physical examination, and mammography, as suggested in the guidelines, are insufficient for the early detection of recurrence and regularly conduct additional imaging and laboratory tests. However, the optimal imaging and laboratory tests to perform in postoperative follow-up of breast cancer patients remain controversial (Lam et al. 2017).

The early detection of local recurrence without distant metastasis has a high probability of being cured (Voogd et al. 2005; Lu et al. 2009). However, for some women, there is limit to the early detection of local recurrence only by physical examination and mammography. Voogd et al. (2005) suggested that recurrence of less than 1 cm after breast-conserving surgery is difficult to detect by physical examination (Kim et al. 2017). Asian (including Korean) and young women have high rates of dense breasts, which reduce mammography sensitivity and produce false negative rates, limiting its usefulness (Kim et al. 2017; Rafferty et al. 2016). Moreover, mammography causes severe pain during testing, and in young women, harm from irradiation may outweigh the benefits (Myers et al. 2015). Equipment-related problems can also lead to poor image quality (Zheng et al. 2018). Therefore, supplemental breast ultrasonography has been conducted recently in addition to mammography; however, additional costs are incurred, and the results may still vary due to differences in the investigator’s level of proficiency (Wojcinski et al. 2011). Therefore, more objective, accurate, and convenient diagnostic and tracking methods are needed to detect local recurrence early after breast cancer surgery.

Recently, we developed a three-protein signature assay called “Mastocheck®” (Bertis, Gyeonggi-do, Korea), which showed 71.6% sensitivity, 85.3% specificity, and 77.0% accuracy in diagnosing early breast cancer (Kim et al. 2019a). Mastocheck® is a breast cancer-specific diagnostic assay based on algorithmic calculations of three plasma protein markers in the blood, carbonic anhydride 1 (CA1), neural cell adhesion molecule L1-like protein (NCHL1), and apolipoprotein C-1 (APOC1), using multiple reaction monitoring (MRM)-based proteomics technique (Kim et al. 2019a; Lee et al. 2015). Mastocheck® was approved for use in humans by the Korean Ministry of Food and Drug Safety in January 2019. The combination of mammography and Mastocheck® showed sensitivity, specificity, and accuracy values of 93.9%, 83.8%, and 90.2%, respectively (Kim et al. 2019b).

The purpose of this study was to evaluate the potential of Mastocheck®, which was developed for early breast cancer diagnosis, in the early detection of recurrence during postoperative follow-up.

## Methods

**Fig. 1:**
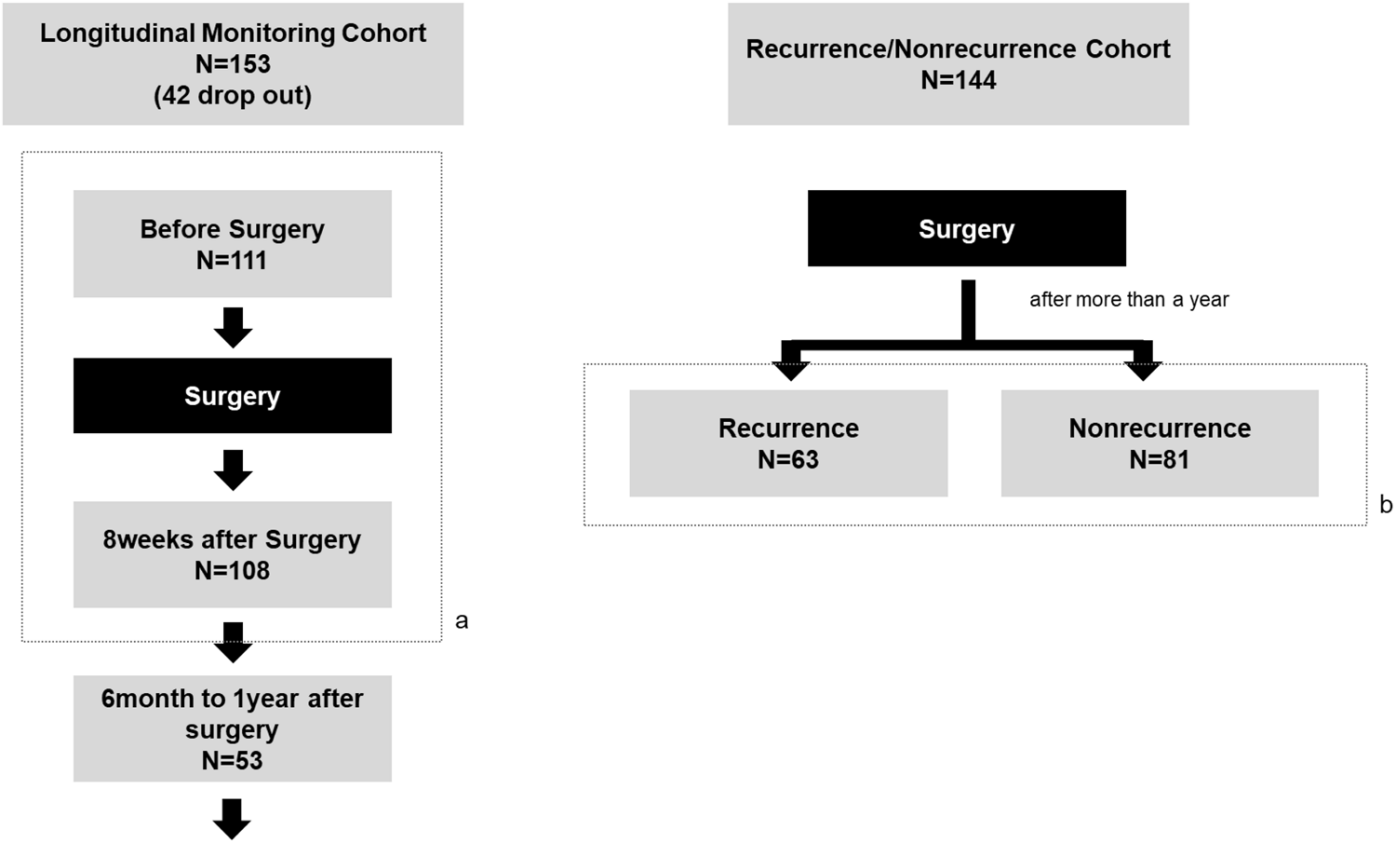
Study design schema. **a** Comparison of Mastocheck® changes before and after surgery. **b** Comparison of Mastocheck® in recurred and non-recurred patients

A total of 255 patients were analyzed. The patients were classified into two cohorts: longitudinal monitoring and recurrence/nonrecurrence cohorts. The scheme of the research design and patient enrollment is shown in Fig. 1.

### Patients and study design

#### Longitudinal monitoring cohort: comparison of changes in Mastocheck® results before and after surgery

Among the patients who underwent surgery at Seoul National University Hospital for invasive breast cancer from August 2018 to December 2020, 153 were prospectively enrolled in this cohort. Among these, 111 patients were finally analyzed after excluding 42 who withdrew their consent during the follow-up period. Blood analyses were performed three times (before surgery, 8 weeks after surgery, and between 6 months and one year after surgery), and a comparative analysis of the values of Mastocheck® and individual proteins at each time point was performed. Of these 111 patients, 108 patients were followed at 8 weeks after surgery was performed in 108 of them. All three serial blood samplings and analyses were performed and 53 patients were followed after 6 months with three times consecutive samplings.

#### Recurrence/nonrecurrence cohort: comparison of Mastocheck® results between recurrence and nonrecurrence patients

This cohort consisted of 63 patients with recurrence and 81 patients without recurrence who underwent surgery between 2005 and 2019 and were followed with  mean follow-up period of around seven years. All 63 patients with recurrence had local recurrence without systemic recurrence, and blood analysis was performed at the time of recurrence diagnosis. Nonrecurrence patients did not have any type of recurrence during follow-up at the time of enrollment in the cohort.

#### Blood collection and three-protein signature blood assay (Mastocheck®) analysis

Blood samples collected in ethylenediaminetetraacetic acid (EDTA) tubes were sent to the laboratory, stored in a deep freezer below − 60 °C, and quantified using a mass spectrometer. The same researcher preprocessed and repeated the experiments two to three times to control the quality of the blood samples. The results of Mastocheck® were obtained through the algorithmic calculations of three plasma protein markers (CA1, NCHL1, and APOC1) developed in previous work. A previous study reported 0.0668 as an optimal cut-off value of Mastocheck® for breast cancer diagnosis, with sensitivity, specificity, and accuracy values of 71.6%, 85.3%, and 77.0%, respectively (Rafferty et al. 2016). Based on this, if the Mastocheck® value was ≥ 0.0668, the sample was considered suspicious for malignancy, and if it was < 0.0668, the sample was considered normal or benign.

### Ethics approval

This study was approved by the Institutional Review Board (IRB) of Seoul National University Hospital (Approval No. D-1905-175-1036), and the study complied with the principles of the Declaration of Helsinki.

### Quantitative protein analysis

Quantitative analysis of the three proteins was performed using commercially available software (Analyst version 1.6, AB SCIEX, Framingham, USA) and reagent solutions (dithioerythritol, iodoacetamide urea, and trypsin). A mass spectrometer (API 5000, AB Sciex, USA [Medical Device License No. Seoul, Korea 10–1245]) was used to perform liquid chromatography–mass spectrometry in MRM mode (Kim et al. 2019a).

### Statistical analysis

First, we identified whether there were significant differences in the included variables between the two groups with and without cancer, according to the Mastocheck® results. Second, changes in Mastocheck® values before and after surgery in patients with breast cancer were evaluated. Clinical pathological information of patients enrolled in the study was collected from the electronic medical records. The differences in protein analysis of recurred and non-recurred patients were also analyzed. IBM SPSS Statistics for Windows, version 21 (IBM Corp., Armonk, NY, USA) and GraphPad Prism 9 were used for statistical analysis.

## Result

### Longitudinal monitoring cohort: comparison of changes in Mastocheck® results before and after surgery

Blood samples were collected three times: before surgery, 8 weeks after surgery, and 6 months to 1 year after surgery. In the preoperative blood analysis of 111 patients, the Mastocheck® value was over the cut-off value in 81 patients and below the cut-off value in 30 patients (73.0% diagnostic sensitivity). The clinicopathologic characteristics of the patients are shown in Table 1.

**Table 1:** Clinicopathologic characteristics of breast cancer patients (longitudinal monitoring cohort)

	Total *N* = 111 (%)	Diagnosed as cancer by Mastocheck *N* = 81 (%)	Diagnosed as normal by Mastocheck *N* = 30 (%)	*p* value
Age (year)	52.78 ± 10.7	52.33 ± 11.08	54 ± 9.69	0.424
BMI	23.85 ± 3.28	24.14 ± 3.54	23.31 ± 2.69	0.207
AJCC stage				
0	5 (4.5)	5 (6.3)	0 (0)	0.197
1	69 (60)	51 (63)	18 (60)	
2	31 (28.2)	20 (25)	11 (36.7)	
3	5 (4.5%)	4 (5)	1 (3)	
T stage				
pTis	5 (4.5)	5 (6.3)	0 (0)	0.669
pT1	76 (69.1)	54 (67.5)	22 (73.3)	
pT2	28 (25.5)	20 (25)	8 (26.7)	
pT3	1 (9)	1 (1.3)	0 (0)	
LN				
pN0	94 (85.5)	69 (86.3)	25 (83.3)	0.784
pN1	11 (10)	7 (8.8)	4 (13.3)	
pN2	3 (2.7)	2 (2.5)	1 (3.3)	
pN3	2 (1.8)	2 (2.5)	0 (0)	
Nucleic grade				
1	5 (4.8)	5 (6.8)	0 (0)	0.472
2	67 (64.4)	47 (63.5)	20 (66.7)	
3	32 (30.8)	22 (29.7)	10 (33.3)	
Histologic grade				
1	13 (12.5)	11 (14.9)	2 (6.7)	0.469
2	65 (62.5)	45 (60.8)	20 (66.7)	
3	26 (25)	18 (24.3)	8 (26.7)	
Subtypes				
HR+/HER2−	82 (78.8)	60 (78.9)	22 (78.6)	0.837
HR+/HER2+	3 (2.9)	3 (3.9)	0 (0)	
HR−/HER2+	9 (8.7)	6 (7.9)	3 (10.7)	
TNBC	10 (9.6)	7 (9.2)	3 (10.7)	
Individual markers				
APOC1	10.97 ± 6.60	9.91 ± 5.49	13.8 ± 8.39	0.015
CA1	14.3 ± 25.7	18.28 ± 29.44	3.548 ± 1.74	< 0.001
NCHL1	1.34 ± 0.76	1.43 ± 0.78	1.10 ± 0.67	0.042

Values are presented as the mean ± standard deviation or number (%)

BMI, body mass index; AJCC, American Joint Committee on Cancer; LN, lymph node; HR, hormone receptor; TNBC, triple negative breast cancer

**Fig. 2:**
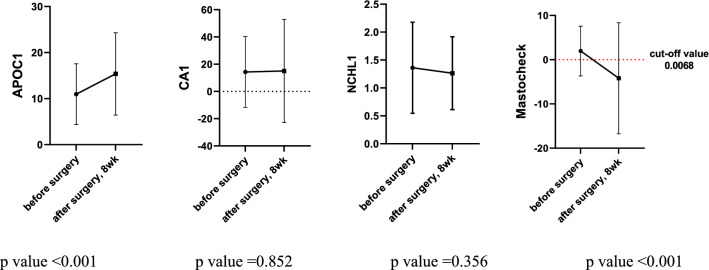
Changes the values of individual markers and Mastocheck® before and 8 weeks after surgery (*N* = 108). APOC1, apolipoprotein C-1; CA1, carbonic anhydride 1; NCHL1, neural cell adhesion molecule L1-like protein

**Fig. 3:**
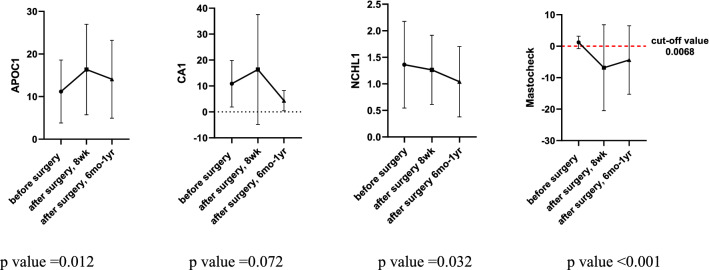
Results of serial analysis before and after surgery up to 1 year (53 paired samples). APOC1, apolipoprotein C-1; CA1, carbonic anhydride 1; NCHL1, neural cell adhesion molecule L1-like protein

Among the 108 patients whose blood analysis was performed at 8 weeks postoperatively, the Mastocheck® value was over the cut-off value in 34 patients (31.5%) and below the cut-off value in 74 (68.5%) patients. Figure 2 shows the changes in the values of Mastocheck® and individual markers before and 8 weeks after surgery. Analysis of individual markers of APOC1, CA1, and NCHL1 showed an increase, a slight increase, and a slight decrease, respectively, at 8 weeks after surgery (*p* value: APOC1 < 0.001, CA1 0.852, and NCHL1 0.356). The value of the three-protein assay Mastocheck® was significantly decreased at 8 weeks after surgery compared to before surgery (*p* < 0.001). For 53 patients, three consecutive blood samplings and analyses were performed: before surgery, 8 weeks after surgery, and 6 months to 1 year after surgery. In a serial analysis of these 53 patients, the value of Mastocheck® was below the cut-off value in 35 patients (66.0%) at 8 weeks after surgery and in 47 patients (88.7%) from 6 months to 1 year after surgery. This indicates that the Mastocheck® value gradually decreases over time after surgery, indicating a normal condition. The results of changes in the values of the three individual markers and Mastocheck® in the serial analysis of 53 patients are shown in Fig. 3. APOC1, which was elevated at 8 weeks after surgery, decreased from 6 months to 1 year after surgery but was still higher than before surgery. CA1, which increased slightly at 8 weeks after surgery, decreased to a lower level than before surgery from 6 months to 1 year after surgery, and NCHL1 showed a tendency to decrease continuously after surgery (*p value*: APOC1 0.012, CA1 0.072, and NCHL1 0.032). The values of the three-protein assay Mastocheck® decreased at 8 weeks after surgery and slightly increased from 6 months to 1 year after surgery but remained significantly lower than before surgery (*p* < 0.001).

### Recurrence/nonrecurrence cohort: comparison of Mastocheck® results between recurrence and nonrecurrence patients

Of the 63 recurrent patients, the values of Mastocheck® were over the cut-off value in 38 patients (60.3%). The median time interval for recurrence was five years and seven months (mean ± standard deviation 7.02 ± 5.08). In the case of CA 15–3, which is being used as a specific blood test for breast cancer, only one in 63 patients with recurrence of this cohort increased. Mastocheck® results were below the cut-off value in 65 (80.2%) out of 81 nonrecurrence patients. The clinicopathologic characteristics of the patients are shown in Table 2.

**Table 2:** Clinicopathologic characteristics of breast cancer patients (Recurrence/nonrecurrence cohort)

	Total *N* = 144 (%)	Recurred *N* = 63 (%)	Non-recurred *N* = 81 (%)	*p* value
Age (year)	56.18 ± 10.11	56.11 ± 10.91	56.09 ± 9.53	0.944
Type of breast surgery				
BCS	100 (69.4)	39 (62.0)	61 (75.3)	0.999
TM	43 (29.9)	23 (36.5)	20 (24.7)	
Unknown	1 (0.7)	1 (1.6)	0 (0)	
Type of axilla surgery				
SLNBx	103 (71.5)	46 (73.0)	57 (70.4)	0.667
ALND	40 (27.8)	16 (25.4)	24 (29.6)	
Unknown	1 (0.7)	1 (1.6)	0 (0)	
AJCC stage (initial)				
0	7 (4.9)	6 (9.5)	1 (1.2)	0.974
1	70 (48.6)	26 (41.3)	44 (54.3)	
2	49 (34.0)	22 (34.9)	27 (33.3)	
3	15 (10.4)	7 (11.1)	8 (9.9)	
Unknown	3 (2.1)	2 (3.2)	1 (1.2)	
T stage				
pTis	7 (4.9)	6 (9.5)	1 (1.2)	0.944
pT1	81 (56.3)	34 (54.0)	47 (58.0)	
pT2	46 (31.9)	19 (30.2)	27 (33.3)	
pT3	6 (4.2)	1 (1.6)	5 (6.2)	
pT4	1 (0.7)	1 (1.6)	0 (0)	
Unknown	3 (2.1)	2 (3.2)	1 (1.2)	
LN				
pN0	93 (64.6)	38 (60.3)	55 (67.9)	0.286
pN1	37 (25.7)	16 (25.4)	21 (25.9)	
pN2	11 (7.6)	7 (11.1)	4 (4.9)	
Unknown	3 (2.1)	2 (3.2)	1 (1.2)	
Subtypes				
HR+/HER2−	82 (56.9)	28 (44.4)	54 (66.7)	0.017
HR+/HER2+	7 (4.9)	4 (6.3)	3 (3.7)	
HR−/HER2+	10 (6.9)	6 (9.5)	4 (4.9)	
TNBC	35 (24.3)	17 (27.0)	18 (22.2)	
Unknown	10 (6.9)	8 (12.7)	2 (2.5)	
Individual markers				
APOC1	11.26 ± 12.14	14.09 ± 17.51	9.05 ± 3.83	0.071
CA1	7.52 ± 8.62	9.73 ± 10.65	5.80 ± 6.16	0.014
NCHL1	0.98 ± 0.89	1.49 ± 1.15	0.59 ± 0.20	< 0.001

Values are presented as the mean ± standard deviation or number (%)

BCS, breast-conserving surgery; TM, total mastectomy; SLNBx, sentinel lymph node biopsy; ALND, axillary lymph node dissection; AJCC = American Joint Committee on Cancer; LN, lymph node; HR, hormone receptor; TNBC, triple negative breast cancer

**Fig. 4:**
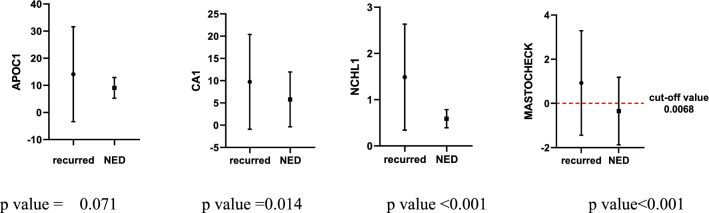
Comparison of individual markers and Mastocheck® values according to recurrence or nonrecurrence among patients observed for more than 1 year after surgery (recurred 63, non-recurred 81). NED, no evidence of disease. APOC1, apolipoprotein C-1; CA1, carbonic anhydride 1; NCHL1, neural cell adhesion molecule L1-like protein

The accuracy of Mastocheck® in the diagnosis of recurrence during follow-up after surgery in this cohort was 71.5%. Figure 4 compares individual markers and Mastocheck® values in recurrence and nonrecurrence patients. All three individual proteins, APOC1, CA1, and NCHL1, showed higher levels in the recurrence group than in the nonrecurrence group (*p* value: APOC1 0.071, CA1 0.014, and NCHL1 < 0.001). Mastocheck® values were over the cut-off value in the recurrence group and below the cut-off value in the nonrecurrence group, which was statistically significant (*p* < 0.001).

## Discussion

Mastocheck® is an algorithm for protein analysis developed for early diagnosis of breast cancer. This study attempted to evaluate whether it would be reasonable to use Mastocheck® as a follow-up test by checking the level of three-protein value after operation and also whether it could detect recurrence after surgery. To this end, it was identified whether the level above the cut-off value before surgery decreased below the cut-off value after surgery.

In this study, we observed initially the value of Mastocheck®, was within the cancer range in 81 of 111 patients (73.0%) before surgery, and after 8 weeks after surgery in 74 of 108 patients (68.5%) became normal range, and 6 month to 1 year after surgery, in 47 of 53 patients (88.7%) became normal range. This suggests that the primary prerequisite for using Mastocheck for follow-up after breast cancer surgery was met. The next step to evaluate the potential of Mastocheck® for the early detection of recurrence during follow-up was to compare its values between the recurrence group and the nonrecurrence group after surgery. In this study, the Mastocheck® value was over the cut-off value in 60.3% of patients with local recurrence and below the cut-off value in 80.2% of patients without recurrence. This suggests that Mastocheck® also met the second requisite as a tumor marker for use during follow-up after surgery.

Since Mastocheck® was initially developed for early breast cancer detection, we focused on evaluating the usefulness of Mastocheck® for the detection of local recurrence rather than regional or systemic recurrence during postoperative follow-up. According to the Early Breast Cancer Trialists’ Collaborative Group (EBCTCG) overview, the 5-year local recurrence risk was 7% in patients after breast-conserving surgery (Clarke et al. 2005). Although many patients with local or regional recurrence will have coexisting distant metastasis simultaneously, for those with isolated local recurrence, long-term survival can be expected through aggressive treatment, including surgery. Recently, Huang et al. (2021) assessed a large breast cancer cohort and reported that patients who underwent salvage surgery after locoregional recurrence showed significantly better 3-year post recurrence survival than those who did not (94.7% vs. 60.7%, *p* = 0.012). The Dutch Study Group on Local Recurrence after Breast Conservation (BORST Group) studied the long-term prognosis of patients with isolated local recurrence after breast-conserving surgery and reported that patients with a local recurrence measuring 1 cm or less had better distant disease-free survival than those with a larger-sized recurrence (Voogd et al. 2005). Lu et al. (2009) reported that patient survival was better when the detection of local recurrence was found earlier through a meta-analysis to establish the impact on survival of early detection of a local recurrence compared to late detection.

Although guidelines recommend mammography alone for the imaging postoperative follow-up of breast cancer patients, early diagnosis of local recurrence with only mammography and physical examination is often difficult. Currently, screening tests for breast cancer diagnosis are mainly imaging tests such as mammography and ultrasonography or magnetic resonance imaging. Mastocheck®, a blood test using proteomics techniques, could be a convenient and reproducible test that overcomes the limitations of imaging tests, especially for women with mammographically dense breasts. Previously, we reported that the combination of mammography and Mastocheck® could increase sensitivity by 30% and accuracy by 15% compared to mammography alone in detecting early breast cancer, resulting in sensitivity, specificity, and accuracy values of 93.9%, 83.8%, and 90.2%, respectively (Kim et al. 2019b). More recently, the combined use of ultrasonography and Mastocheck® showed significantly improved diagnostic specificity and positive predictive value for breast cancer diagnosis compared to ultrasonography alone, even in asymptomatic women, women with dense breasts, or those with normal/benign mammographic findings, showing that Mastocheck® is an effective tool that can be used with ultrasound to improve diagnostic specifications and reduce false-positive findings and unnecessary biopsies. Utilizing the Mastocheck® value with ultrasonography ncreased the AUC from 0.67 to 0.81 and the specificity from 35.6 to 64.4% without loss in sensitivity. The biopsy rate was significantly decreased from 79.3 to 72.1% (Ha et al. 2022). These results suggest that using Mastocheck® as an adjunct, along with imaging tests such as mammography and ultrasonography during follow-up after breast cancer surgery, can aid in the early detection of local recurrence. The results of the present study successfully demonstrated that Mastocheck® could be helpful in the detection of local recurrence during follow-up after breast cancer surgery.

Many studies have shown that CA15-3, widely used as a breast cancer-specific biomarker, is not useful for early diagnosis as a single marker due to its low sensitivity and specificity and because it showed no significant correlation with cancer metastasis during follow-up (Rasmy et al. 2016; Elfagieh et al. 2012). The tumor marker CA15-3 has been studied in the primary diagnosis of breast cancer and in metastatic settings. It has been found to be elevated in breast cancer in stage I in 9%, stage II in 19%, stage III in 38%, and stage IV (distant metastatic disease) in 75% (American Society of Clinical Oncology 1996). In another study, the CA15-3 increase in patients confirmed to have recurrence was approximately 36%, which was low in sensitivity, and even in those with distant metastasis in the liver or bone, it was only approximately 48%, suggesting that CA15-3 has limitations in confirming early local recurrence as a single test (Kokko et al. 2002). In our study, only one in 111 patients in the longitudinal monitoring cohort had an increased preoperative CA15-3 level. All 63 patients with recurrence enrolled in the recurrence/nonrecurrence cohort had local recurrence, and CA15-3 was in the normal range in all of them. This also suggested that CA15-3 is not sensitive for the early detection of recurrence. In our study, the diagnostic sensitivity of Mastocheck® in recurrence patients was 60.3%, which is superior to that of CA15-3. This could be the basis for the validation of Mastocheck® as a follow-up test after surgery.

Analysis of the individual markers showed that CA1 and NCHL1 levels increased while APOC1 levels decreased in breast cancer (Li et al. 2019; He et al. 2013; Sun et al. 2016). Therefore, after cancer tissue is removed from the body after surgery, CA1 and NCHL1 decrease, and APOC1 increases. In this study, CA1 was slightly increased, NCHL1 was decreased, and APOC1 was increased at 8 weeks after surgery compared to before surgery. Only APOC1 showed statistically significant changes in individual marker changes, reflecting that APOC1 significantly influences the overall Mastocheck® changes immediately after surgery. APOC1 is a lipid-related protein marker, and it is necessary to study whether it is affected by changes in the lipid profile of patients receiving aromatase inhibitors after surgery.

Proteomics can determine the level of protein in the cell, which helps explain where they are located. The effects of the cell environment can also be observed. In other words, it allows us to see how the levels of protein change and how cells react (Wilhelm et al. 2014). For this reason, researchers have tried to develop biomarkers using proteomics techniques. Mastocheck® is the result of years of experimentation with various designs to confirm the usefulness of breast cancer diagnosis. Mastocheck® was developed for the early diagnosis of breast cancer using 1,129 stored blood samples analyzed through quantification and optimization processes. In addition, correlation evaluation with other cancers confirmed it to be a unique marker for breast cancer diagnosis. Various methods, such as correlation evaluation with anesthesia, have been conducted to develop an objective and universal diagnostic marker for breast cancer diagnosis (Kim et al. 2019a).

In conclusion, our data suggest the possibility of Mastocheck® as a blood marker tool for the early detection of recurrence during postoperative follow-up, based on normalization in 88.7% of patients one year after surgery and 71.5% accuracy for locally recurrence patients. The limitations of this study are that it is an ongoing study, and it was difficult to generalize the interpretation to all patients with breast cancer due to the small number of subjects analyzed and the short duration of follow-up. Despite these limitations, proteomic biomarkers could provide a new paradigm in the clinic that is useful not only for early diagnosis but also for follow-up after treatment.

## Abbreviations

APOC1: Apolipoprotein C-1
CA1: Carbonic anhydride 1
NCHL1: Neural cell adhesion molecule L1-like protein
IRB: Institutional Review Board
LC‒MS/MS: Liquid chromatography–mass spectrometry
MRM: Multiple reaction monitoring
